# Development of Gefitinib-Loaded Solid Lipid Nanoparticles for the Treatment of Breast Cancer: Physicochemical Evaluation, Stability, and Anticancer Activity in Breast Cancer (MCF-7) Cells

**DOI:** 10.3390/ph16111549

**Published:** 2023-11-02

**Authors:** Ibrahim A. Aljuffali, Md. Khalid Anwer, Mohammed Muqtader Ahmed, Ahmed Alalaiwe, Mohammed F. Aldawsari, Farhat Fatima, Shahid Jamil

**Affiliations:** 1 Department of Pharmaceutics, College of Pharmacy, King Saud University, P.O. Box 2457, Riyadh 11451, Saudi Arabia; ialjuffali@ksu.edu.sa; 2 Department of Pharmaceutics, College of Pharmacy, Prince Sattam Bin Abdulaziz University, Al-kharj 11942, Saudi Arabia; mo.ahmed@psau.edu.sa (M.M.A.); a.alalaiwe@psau.edu.sa (A.A.); moh.aldawsari@psau.edu.sa (M.F.A.); f.soherwardi@psau.edu.sa (F.F.); 3 Department of Pharmacy, College of Pharmacy, Knowledge University, Erbil 44001, Iraq; shahidjamil07@gmail.com

**Keywords:** gefitinib, lipid, surfactant, solid lipid nanoparticles, stability, breast cancer cell, MTT assay, anticancer

## Abstract

In the current study, the toxic effects of gefitinib-loaded solid lipid nanoparticles (GFT-loaded SLNs) upon human breast cancer cell lines (MCF-7) were investigated. GFT-loaded SLNs were prepared through a single emulsification–evaporation technique using glyceryl tristearate (Dynasan™ 114) along with lipoid^®^ 90H (lipid surfactant) and Kolliphore^®^ 188 (water-soluble surfactant). Four formulae were developed by varying the weight of the lipoid™ 90H (100–250 mg), and the GFT-loaded SLN (F4) formulation was optimized in terms of particle size (472 ± 7.5 nm), PDI (0.249), ZP (−15.2 ± 2.3), and EE (83.18 ± 4.7%). The optimized formulation was further subjected for in vitro release, stability studies, and MTT assay against MCF-7 cell lines. GFT from SLNs exhibited sustained release of the drug for 48 h, and release kinetics followed the Korsmeyer–Peppas model, which indicates the mechanism of drug release by swelling and/or erosion from a lipid matrix. When pure GFT and GFT–SLNs were exposed to MCF-7 cells, the activities of p53 (3.4 and 3.7 times), caspase-3 (5.61 and 7.7 times), and caspase-9 (1.48 and 1.69 times) were enhanced, respectively, over those in control cells. The results suggest that GFT-loaded SLNs (F4) may represent a promising therapeutic alternative for breast cancer.

## 1. Introduction

Gefitinib (GFT) is a small molecule that belongs to the quinazoline family with molecular formula C_22_H_24_ClFN_4_O_3_, and the IUPAC name is 4-Quinazolinamine, N-(3-chloro-4-fluorophenyl)-7-methoxy-6-[3-(4-morpholinyl)propoxy]. The molecular weight of GFT is 446.9 g/mol, with a partition coefficient (logP) of about 3.5, which indicates that it has a moderate lipophilicity and can cross cell membranes [1,2]. It is a pale yellow powder, sensitive to light, and prone to degradation upon exposure to UV radiation. It is slightly soluble in water (0.1 mg/mL) but more soluble in organic solvents such as methanol, DMSO, ethanol, and acetone [3,4].

GFT was first developed by AstraZeneca and was approved by the U.S. Food and Drug Administration (FDA) in May 2003 [5]. GFT is used in the targeted therapy category due to its ability to block the epidermal growth factor receptor (EGFR), which is overexpressed in many types of cancer and promotes the growth and spread of cancer cells. As an EGFR tyrosine kinase inhibitor, GFT disrupts EGFR signaling and thus leads to the inhibition of cell proliferation, migration, and survival and the inducement of apoptosis in cancer cells. Reported side effects of GFT include skin rash, diarrhea, and liver toxicity [6,7,8]. In addition to its use in non-small-cell lung cancer, GFT has also been investigated for the treatment of other types of cancer, such as breast cancer, ovarian cancer, and head and neck cancer [9,10]. It has been suggested that GFT alone or in combination can be used in breast cancer treatment. MCF-7 is a type of breast cancer cell that expresses EGFR [11,12,13,14]. GFT was found to have a synergistic impact with the tamoxifen treatment, inhibiting the proliferation of endocrine-resistant MCF-7 breast cancer cells via lowering AKT and MAPK phosphorylation [15,16].

A study on the effects of GFT in combination with docetaxel, a commonly used chemotherapy drug, on MCF-7 cells found that the combination of GFT and docetaxel resulted in greater antiproliferative effects than either drug alone, suggesting that combining GFT with other chemotherapy drugs may enhance anticancer efficacy [13]. However, its use in these cancers is not yet well established and is still being studied in clinical trials.

To improve the bioavailability and therapeutic efficacy of GFT, researchers have been investigating various drug delivery systems. Nanoliposomes were developed for GFT to improve the solubility, stability, bioavailability, and efficacy of GFT in the treatment of lung cancer [15]. Using ionic gelation, Gupta et al. prepared polymeric nanoparticles to encapsulate drugs and improve their delivery and release at the target tumor cells more precisely [16]. In another study, PEGylated solid lipid nanoparticles were developed as a promising approach for lymphatic delivery of GFT [17]. Similarly, studies on GFT-loaded nanomicelles, self-assembled structures made of surfactants to target lung cancer stem cells, have the potential significantly to impact cancer therapy [18].

The development of stable GFT-loaded SLNs and further investigation of their potential application in the treatment of breast cancer are the goals of this study. Lipid Dynasan™ 114, lipid surfactant (lipoid 90H), and water-soluble surfactant (Kolliphore^®^ 188) were used to create the GFT-loaded SLNs. The developed SLNs were tested against the MCF-7 breast cancer cell lines. GFT represents an established in vitro experimental model to study cytotoxicity effects, which is why the MCF-7 cell lines were chosen [19]. If the newly synthesized formulation demonstrates the desired cytotoxic action, the results of this study may eventually be used to treat breast cancer.

## 2. Results and Discussion

### 2.1. Particle Characterization and Drug Encapsulation

Among the four SLNs, GFT-loaded SLNs (F4) (Lipoid 90H, 250 mg) had better particle properties (Table 1). In general, the particle size varied, ranging from 378 to 472 nm. They have the benefit of allowing the drug entrapped within SLNs to pass through physiological drug barriers. Endocytosis allows for the passage of particles smaller than 500 nm through the epithelial cell membrane [20,21,22]. It is obvious that an increase in the concentration of lipid surfactant in the formulation results in an increase in particle size. It was found that the zeta potential increases with an increase in the Lipoid 90H amount in the formulation. As a result, a zeta potential of −15.2 mV was found to be more stable for GFT-loaded SLNs (F4) than for other formulations. At various lipid concentrations, the entrapment efficiency (EE) of GFT was also investigated in SLNs. It was found that the quantity of lipid surfactant affected the extent of GFT entrapment from 64.07 to 83.18%.

### 2.2. Thermal Studies

**Figure 1:**
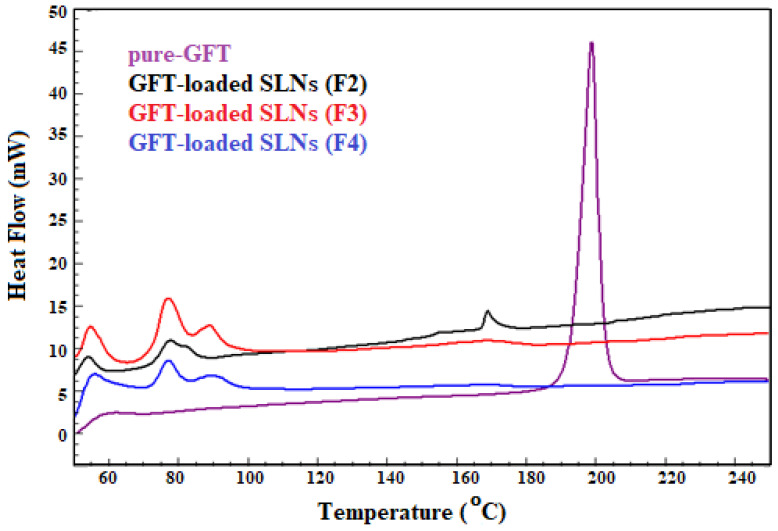
Characterization of pure GFT and GFT-loaded SLNs (F1-F4) using DSC spectra.

A comparison of the thermal spectra of pure GFT and GFT-loaded SLNs (F2–F4) is shown in Figure 1. The DSC spectra of pure GFT showed a distinct endothermic peak at 199 °C, which was consistent with the literature [23,24,25]. GFT-loaded SLNs (F2) showed a reduced endothermic peak of GFT with minor shifting, while GFT-loaded SLN (F3) and SLN (F4) showed a complete disappearance of GFT peaks, demonstrating that there was no interaction or incompatibility between the drug and the excipients used, which is consistent with the diluting effect caused by the presence of lipids.

### 2.3. In Vitro Release Studies

**Figure 2:**
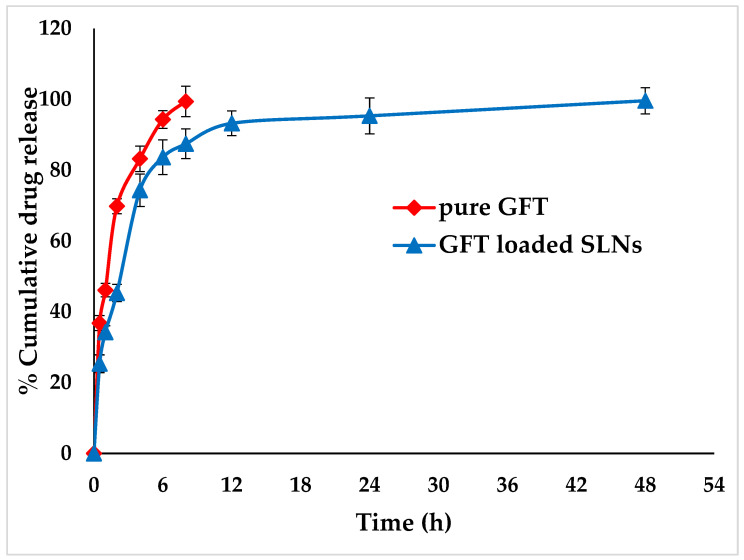
In vitro release of pure GFT and optimized GFT-loaded SLNs (F4).

SLNs are composed of dynasan lipids that can encapsulate GFT. These nanoscale particles were stabilized by a combination of poloxomer and lipoid stabilizers. Prepared GFT-loaded SLNs (F4) could serve as sustained drug carriers and also as drug stabilizers (Figure 2). The release interpretation of the pure drug at pH 7.4 demonstrates that initially, there was no release. Over time, however, GFT release increased gradually at 0.5 h (36.8%). The release continued to increase reaching 36.8%, 69.8%, and 83.2% at 1, 2, and 4 h, respectively. The drug release further progressed to 94.3% at 6 h and 99.4% at 8 h. The SLN formulation appears to have a sustained release rate compared to pure GFT. Further, it appears that the release reached a plateau or near-complete release at later time points. Since Dynasan™ 114 is a solid lipid with a high melting point, it can provide a sustained release effect by delaying the diffusion of the drug molecules from the Lipid Matrix. Lipid Dynasan™ 114 is a synthetic lipid used in SLNs [26]. Lipid surfactant (e.g., Lipoid 90H) could be instrumental in improving GFT drug release over a prolonged period by promoting its solubility in the lipid matrix and sustaining its release. The presence of Lipoid 90H at the particle surface helps prevent particle aggregation and also contributes to a controlled drug release profile by inhibiting burst release and promoting sustained release over time [27]. Water-soluble surfactants such as Kolliphore^®^ 188 are used in combination with lipid surfactants to stabilize SLNs. Kolliphore^®^ 188 is a block copolymer composed of hydrophilic poly(ethylene oxide) (PEO) and hydrophobic poly(propylene oxide) (PPO) segments. The PEO chains can adsorb onto the surface of SLNs, creating a stearic barrier similar to the lipid surfactants, avoiding agglomeration and keeping particles small in size, thereby improving the effective surface area for dissolution of the drug substance from the carriers [28]. The presence of Kolliphore^®^ 188 can alter the release kinetics by regulating the diffusion of the drug through the surfactant layer. Additionally, the hydrophilic nature of Kolliphore^®^ 188 can enhance the release of hydrophilic drugs by facilitating their dissolution and diffusion through the aqueous media [29,30]. Using a combination of lipid Dynasan™ 114, Lipoid 90 H, and Kolliphore^®^ 188 in the preparation of GFT-loaded SLNs exerts a synergistic influence on the drug release profile.

The release kinetics data of GFT-loaded SLNs (F4) show the correlation coefficients (R^2^ values) for different drug release kinetic models at pH 7.4. The zero-order model suggests that the drug is released at a constant rate over time at an R^2^ value of 0.714, which suggests that the GFT released was independent of the drug concentration. The first-order model assumes an exponential decay in GFT release over time, with an R^2^ value of 0.878, which indicates a better fit to the first-order model compared to the zero-order model. The Higuchi model describes drug release based on Fickian diffusion, where the release rate is proportional to the square root of time. The R^2^ was found to be 0.715, indicating a moderate fit to the Higuchi model. Conversely, the R^2^ of SLNs was found to be 0.909, indicating a reasonable fit to the Korsmeyer–Peppas model. The release exponent (n value) falls between 0.45 and 0.89; it indicates a combination of diffusion and other factors, such as swelling, erosion, or relaxation of the matrix. The n value observed by us was 0.309. The postulated mechanism of drug release may involve swelling and/or erosion of the lipid matrix because a higher R2 was seen in the Korsmeyer–Peppas model and along with an n value of less than 0.3 from optimized SLNs [31].

### 2.4. Morphology

**Figure 3:**
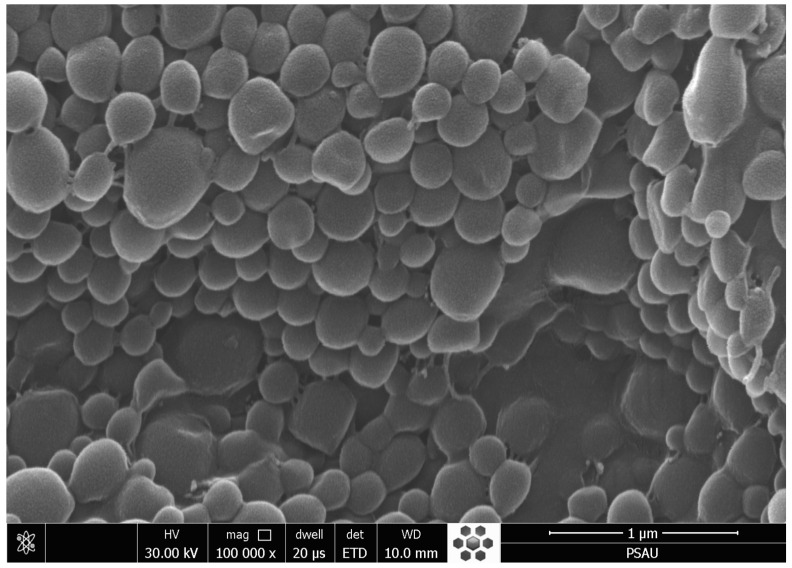
SEM characterization of optimized GFT-loaded SLNs (F4).

The scanning electron microscopy (SEM) images of optimized GFT-loaded SLNs (F4) appeared smooth, spherical, and aggregated. The size observations of these particles conform with their DLS data. The lyophilizing aqueous SLN dispersions may encourage nanoparticle aggregation, particularly when freeze drying is performed without the application of a cryoprotectant (Figure 3).

### 2.5. Stability Studies

The size, polydispersity index (PDI), zeta potential (ZP), and EE of the optimized GFT-loaded SLNs (F4) were studied at different time intervals (1, 2, and 3 months) after storage at 25 ± 1 °C and 37 ± 1 °C. The results are tabulated in Table 2. No significant changes were observed (*p* < 0.05) in the size, PDI, ZP, and EE at either storage temperature. The EE analysis revealed that the GFT was present at a 77.8% level after 3 months, even at 37 °C, indicating that the GFT-loaded SLNs (F4) were chemically stable and did not undergo any drug alteration or decomposition. According to the results of the drug content study, the probe sonication procedure used to prepare the SLNs could not have affected the drug’s chemical stability. As a result, the developed GFT-loaded SLNs can be kept for up to three months at room temperature without significantly losing their drug content or other physical properties.

### 2.6. MTT Assay on Breast Cancer Cell Lines

**Figure 4:**
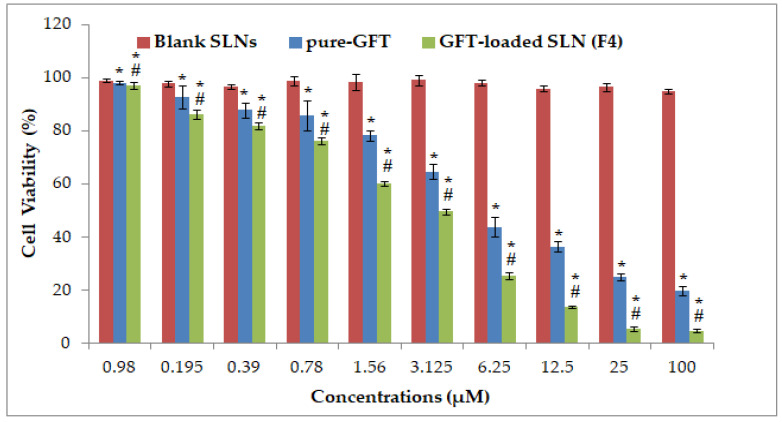
Cytotoxicity profile of blank SLNs, pure GFT, and optimized GFT-loaded SLNs (F4) at varying concentrations (0.98 to 100 µg/mL). “Data are expressed as mean ± SD (n = 3). Significant difference (* *p* < 0.05) was observed in blank SLNs vs. pure GFT; pure GFT vs. F4; and (^#^ *p* < 0.001) in blank SLNs vs. F4”.

Figure 4 illustrates the cytotoxicity of pure GFT and GFT–SLNs towards MCF-7 cells. The IC50 of pure GFT and GFT–SLNs against MCF-7 were 4.0822 µM and 2.7814 µM, respectively. The GFT-loaded SLNs (F4) indicated a significant reduction in cell viability (100.93%, 85.98%, 82.00%, 76.16%, 60.19%, 49.73%, 25.642%, 13.817%, 5.565%, and 4.82% at 0.098, 0.195, 0.39, 0.78, 1.56, 3.125, 6.25, 12.50, 25, and 100 µM, respectively) in comparison to pure GFT (98.214%, 92.866%, 87.898%, 85.884%, 78.28%, 64.79%, 43.93%, 36.53%, 25.08%, and 19.84% at 0.098, 0.195, 0.39, 0.78, 1.56, 3.125, 6.25, 12.50, 25, and 100 µM, respectively), as depicted in Figure 4, against MCF-7 cells. In the MTT assay using MCF-7 cells, the cytotoxic effect of GFT-loaded SLNs was significantly (*p <* 0.001) increased in comparison to pure GFT and blank SLNs. The GFT-loaded SLNs improve GFT’s anticancer activity in MCF-7 cells, which is thought to be due to the increased bioavailability and cellular uptake of drugs in the SLNs. As a result, the use of SLNs with GFT could be a replacement for the current method of treating human cancer with pure GFT.

### 2.7. Morphological Changes in MCF-7 Cells Treated with GFT-Loaded SLNs

**Figure 5:**
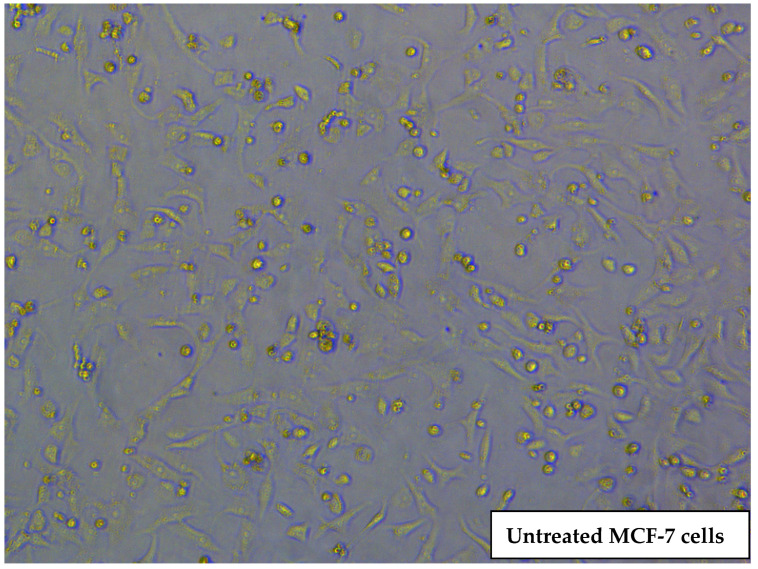

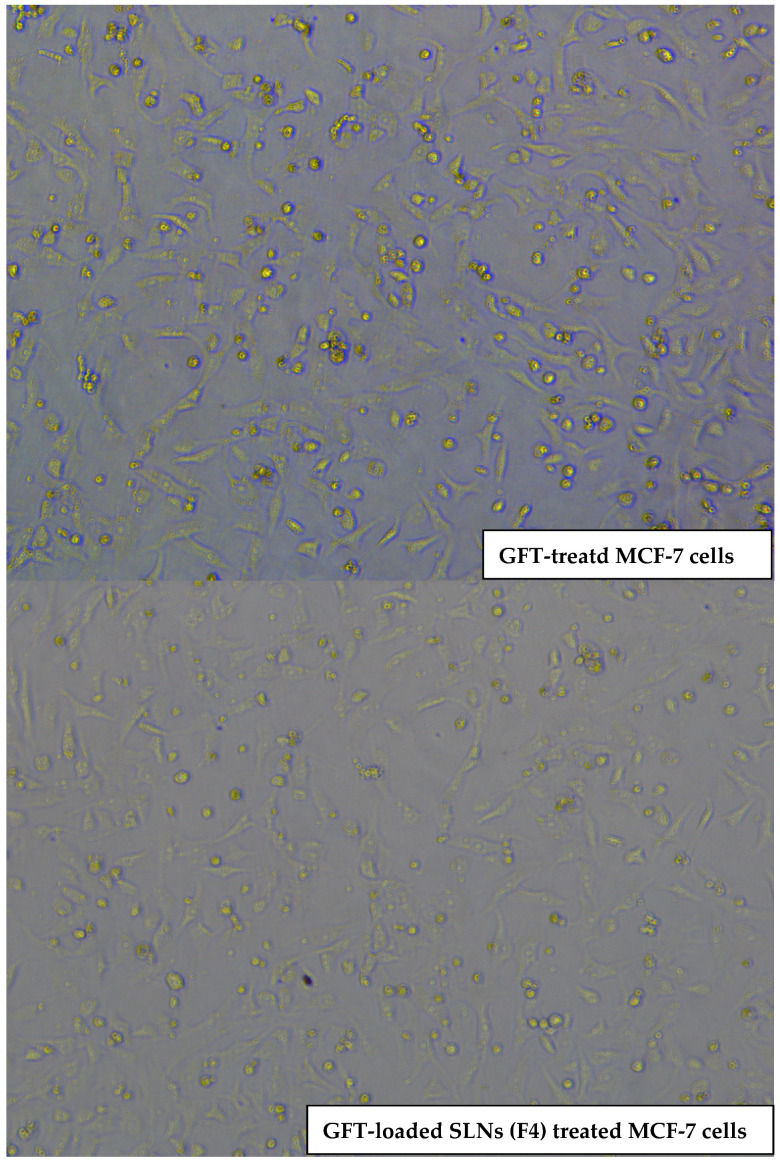
Morphological changes in MCF-7 treated cells. (magnification 200×).

Figure 5 shows the morphological changes of MCF-7 cell lines after treatment with control, pure GFT, and GFT-loaded SLNs (F4). Compared to the control and pure GFT cells, cells treated with GFT-loaded SLNs showed the greatest amount of cell death after 24 h of incubation. Shrinkage and reduction in the number of adherent viable cells and increase in floating dead cells were observed in MCF-7 cells treated with pure GFT and GFT-loaded SLNs (F4). This corresponds to the data from the MTT assay. The morphological changes are caused by damage to the cell organelles. Untreated MCF-7 cells typically exhibit an epithelial-like morphology; cells appear to be densely packed together. Individual MCF-7 cells often have a round or polygonal shape. When cultured at high density, MCF-7 cells tend to be tightly packed together, forming cohesive cellular clusters or islands. GFT-treated MCF-7 cells exhibit a reduced size compared to untreated cells. This shrinkage can result from various phenomena, such as cytoskeletal rearrangements or cellular dehydration. Treated cells display irregular or elongated shapes, differing from the typical round or polygonal morphology of untreated MCF-7 cells. Also treated cells exhibited a disruption in cell–cell junctions, leading to a loss of the well-defined cobblestone-like appearance. GFT-treated cells appear more scattered. GFT-loaded SLNs reduced the proliferation of MCF-7 cells, resulting in fewer cells compared to pure GFT-treated cells and untreated MCF-7 cells, as observed under a microscope. Cell-to-cell adhesion properties of MCF-7 cells were also found to be modified with F4 treatment. This may result in altered cell clustering, a loss of tight junctions, or changes in the formation of adherens junctions. GFT treatment with SLN F4 may induce programmed cell death, such as apoptosis, in MCF-7 cells.

### 2.8. ELISA Assay of P53, Caspase-3, and Caspase-9

**Figure 6:**
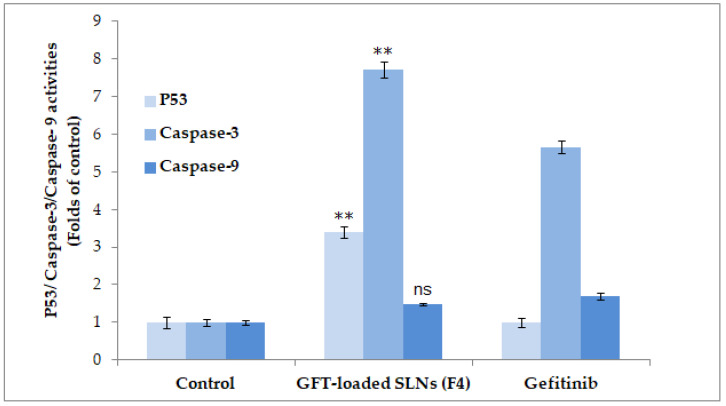
Effects of GFT-loaded SLNs on the level of p53, caspase-3, and caspase-9. Data were observed to show significant difference (** *p* < 0.05) in GFT-loaded SLNs (F4) vs. control and pure GFT (P53 and caspase-3) and non-significant difference (*p* > 0.05) between GFT-loaded SLNs (F4) and pure GFT (caspase-9).

Apoptosis, or programmed cell death, is mostly carried out through activated caspase-3 and the activation of p53 [32,33,34]. It selectively disassembles many intracellular components without harming or inflaming the neighboring normal cells. T chemotherapy-induced cell death and cancer cell apoptosis have been linked to p53, caspase-3, and caspase-9 activities. In this study, p53 expression in pure GFT and GFT–SLN groups was significantly increased compared to the untreated group (*p* < 0.05), indicating that GFT-loaded SLN (F4) > pure GFT has many characteristics. Strong apoptotic activity was exhibited when pure GFT and GFT–SLNs were exposed to MCF-7 cells; the activities of p53 (3.4 and 3.7 times), caspase-3 (5.61 and 7.7 times), and caspase-9 (1.48 and 1.69 times) were enhanced in those samples, respectively, compared to the control cells (Figure 6). The greater efficacy of GFT in SLNs raises a potential explanation for the triggering of apoptosis in cancer cells.

## 3. Materials and Methods

### 3.1. Materials

The GFT was bought from “Mesochem Technology in Beijing, China”. Glyceryl trimyristate (Dynasan 114), Kolliphore^®^ 188, Lipoid 90H, and dichloromethane were bought from “Sigma Aldrich, St. Louis, MO, USA”. All solvents/chemicals and purified water (MilliQ) used throughout the study were of analytical grade.

### 3.2. Preparation of GFT-Loaded SLNs

Four batches of GFT-loaded SLNs (F1-F4) were prepared by keeping a constant amount of pure drug (100 mg), lipid (Dynasan™ 114, 100 mg), and surfactant (Kolliphore 188, 0.5% *w*/*v*) and varying the amount of Lipoid 90H (as 100, 150, 200, and 250 mg) following the reported single emulsification technique [35,36,37]. Briefly, GFT (100 mg) was dissolved in a previously prepared solution of lipid Dynasan 114 and lipoid 90H in 5 mL of dichloromethane. On the other hand, the aqueous phase containing Kolliphore^®^ 188 (20 mL, 0.5%, *w*/*v*) was prepared separately. By adding the aqueous phase with a syringe at a rate of 0.3 mL/min while using a probe sonicator “(model CL-18, Fisher Scientific; Hampton, NH, USA)” for 3 min at 65% W voltage efficiency and 5 sec ON/OFF cycles, the lipid phase was emulsified. On a magnetic stirrer, the organic solvent was evaporated for six hours. GFT-loaded SLNs were collected after high-speed (15,000 rpm) centrifugation “(HermleLabortechnik, Z216MK, Wehingen, Germany)”, washed thrice with deionized water, and freeze dried. Collected drug-loaded SLNs were then lyophilized “(Millrock Technologies, Kingston, NY, USA)” and preserved for further studies. Table 3 lists the composition of four developed GFT-loaded SLNs (F1–F4). The percent entrapment efficiency (%EE) of developed formulations was measured indirectly by separating aqueous free drug in supernatant after high speed centrifugation (10,000 rpm). The amount of drug that was free was determined using UV spectrophotometry at 331 nm [38], and EE% was calculated using the formula shown below [39]:

$$EE\left(\%\right)=\frac{Total\;GFT-free\;GFT}{Total\;GFT}\times 100$$

### 3.3. Particle Characterization and Drug Encapsulation

The mean particle size, PDI, and ZP of the GFT-loaded SLN preparation were evaluated through the dynamic scattering light (DLS) method using a Malvern zetasizer (ZEN-3600, Malvern Instruments Ltd., Worcestershire, UK). The mean particle size and PDI were determined using an appropriately diluted (200 times) fresh colloidal dispersion placed into a plastic disposable cuvette. The zeta potential of the nanoparticles was measured to determine their surface charge, which also denotes surface modifications. The procedure for ZP measurement was the same as that for mean particle size and PDI, except that a glass electrode cuvette was used.

### 3.4. Thermal Studies

Differential scanning calorimetry was used with the Korean-made Scinco-N650 instrument for the thermal characterization of pure GFT and GFT-loaded SLNs (F2–F4). In addition, 5 mg of the test samples was compressed in an aluminum pan and placed in a sample holder for comparison with the reference sample (an empty aluminum pan). With nitrogen gas flowing continuously at a rate of 15 mL/min, scanning was carried out in the temperature range of 50 to 250 °C.

### 3.5. In Vitro Release Studies

In vitro release assessments of pure GFT and optimized GFT-loaded SLNs (F4) have been completed in a phosphate buffer (pH 7.4). Accurately weighed amounts of pure GFT (10 mg) and GFT-loaded SLNs (equivalent to ten mg of natural GFT) were suspended in a dissolution medium (10 mL) and poured into a dialysis bag “(molecular weight reduce off at 12,000 Daltons)”. After that, the dialysis bag was put into a beaker with 40 mL of the appropriate medium and kept on a biological shaker “(LBS-030S-Lab Tech, Jeju, Republic of Korea)” at 100 rpm and 37 °C. The sink condition was maintained by taking 1 mL of the sample at regular intervals and replacing it right away with the media. The collected samples were then appropriately diluted before being tested for drug content using UV spectroscopy at a maximum wavelength of 331 nm [38]. Furthermore, by incorporating the drug release findings into various mathematical kinetics models, the GFT drug release mechanism from the GFT-loaded SLNs (F4) at pH 7.4 could be estimated [40,41].

### 3.6. Morphology

The gold-sputter technique was used to characterize the morphology of optimized GFT-loaded SLNs (F4) using “SEM (Zeiss EVO LS10, Cambridge, UK)”. Using the “Q-150R Sputter Unit” from “Quorum Technologies Ltd. (Laughton, East Sussex, UK)” for 60 s at 20 mA in an argon atmosphere, the freeze-dried SLNs were coated with gold. Imaging was conducted using an accelerating voltage of 30 kV and a magnification of 10–50 KX.

### 3.7. Stability Studies

The stability of optimized GFT-loaded SLNs (F4) was assessed in terms of size, PDI, ZP, and EE% by following previously published reports [42,43]. This was accomplished by simply packaging 10 mg of freeze-dried GFT-loaded SLNs (F4) in an amber glass vial and storing it at 25 °C and 37 °C for three months. The changes in the size, PDI, ZP, and drug content were recorded periodically (i.e., the seventh day, first month, third month, and sixth month). To assess these parameters, the previously stored GFT-loaded SLNs (F4) were re-suspended in PBS (pH 7.4).

### 3.8. MTT Cytotoxicity Studies

#### 3.8.1. Cell Growth

The MCF-7 breast cancer cells were bought from the American Type Cutler Collection (ATCC) in Manassas, Virginia, in the United States. Cells were cultured in “Dulbecco’s modified Eagle’s medium (DMEM) (UFC Biotech, Riyadh, Saudi Arabia)” using tissue culture flasks with an area of 50 cm^2^ in a humidified incubator at 37 °C, 5% CO_2_. Supplementary media contained 10% fetal calf serum “(Alpha Chemika, Mumbai, India)”, 1% penicillin mixture (100 units/mL), streptomycin (100 μg/mL), and 1% L-glutamic acid. The MTT detection kit was bought from “Sigma Aldrich (St. Louis, MO, USA)”. In 96-well cell culture plates containing DMEM, cells were seeded.

#### 3.8.2. MTT Assay on Breast Cancer Cell Lines

The anticancer potentials of GFT–SLNs and the pure drug were compared through the MTT assay using MCF-7 cells. Due to mitochondria-mediated apoptosis, the MTT test largely determines the viability of cells. In this test, 3-(4,5-dimethylthiazol-2-yl)-2,5-diphenyltetrazolium bromide, a water-soluble dye, is enzymatically converted to insoluble formazan, and the amount of formazan reveals the relative viability of the cell [44,45,46,47]. According to the experimental protocol, cell viability was determined using trypan blue dye before the experiment, and cell viability was found to be 95%. Cells were treated with the formulation when they were between passages 10 and 12. Briefly, 100 µL of medium was used to seed the cells in a 96-well microplate and kept overnight. The suspensions of blank SLNs, pure GFT, and GFT–SLNs (F4) were prepared using the medium as a diluent, while the GFT concentration ranged from 0.98 to 100 µg/mL [48]. The medium was taken out; the cells were exposed to the GFT formulations; and a control experiment (cells only receiving medium) was also performed. The drug-exposed cells were treated with 100 µL of MTT solution (5 mg/mL in PBS) after 48 h. Fresh medium devoid of medication was substituted for the media for the growing cells, and the cells were then incubated for an additional 4 h at 37 °C after being treated with 20 µL of MTT. The culture fluid was then discarded, and MTT crystals were then dissolved for 15 min at working temperature in a solution of DMSO, acetic acid, and sodium dodecyl sulfate (99.5 mL, 0.6 mL, and 10 g). Then, using a spectrophotometric microplate reader (Synergy HT, BioTek Instruments, Winooskim, VT, USA), the optical density (OD) of this solution was determined at 570 nm. Equation (1) was then used to calculate the cell viability (%). The data were plotted as a function of drug concentration (g/mL) versus cell viability (%), with the values normalized to those obtained for viable control cells (considered as 100%). The IC50 values were calculated using log(inhibitor) against the normalized response curve.

(1) $$\%\;Cell\;viability=\frac{OD\;of\;sample-OD\;of\;blank}{OD\;of\;control-OD\;of\;blank}\times 100$$

#### 3.8.3. Morphological Changes in MCF-7 Cells Treated with GFT-Loaded SLNs

For morphological studies through phase contrast microscopy, the MCF-7 cells were seeded in 6-well plates (1 × 10^5^ per well) containing DMEM and were incubated at 37 °C in 5% CO_2_ for 24 h. Thereafter, cells were treated with the GFT–SLN formulations containing 3.21 μg/mL of GFT and were incubated for 48 h. Then, cells were observed using phase contrast microscopes (Olympus CLX 41, Olympus Corporation, Tokyo, Japan) for changes in the cell structure/apoptosis, and the photographs were recorded.

#### 3.8.4. ELISA Assay of P53, Caspase-3, and Caspase-9

Following the manufacturer’s instructions and published studies [34,49], the activities of p53, caspase-3, and caspase-9 were assayed through ELISA. The MCF-7 cells were seeded in 96-well plates (5 × 10^4^ cells/well), followed by treatment with pure GFT and GFT–SLNs. The pure GFT, GFT–SLNs, and untreated control cells were subsequently given time to acclimate in 96-well plates at room temperature. Then, 100 µL of the p53, caspase-3, and caspase-9 reagents were added to each of the corresponding wells of the plate that was pre-filled with 100 µL of culture media. After the treatment, the plates were covered, stirred at 500 rpm for 1–2 min, and then incubated at room temperature for 1 h. Finally, the OD was measured using a microplate reader at the wavelength of 405 nm.

## 4. Conclusions

In recent years, SLNs with different sizes and characteristics have been developed and extensively analyzed. SLNs are highly effective in enhancing the therapeutic effects and minimizing the side effects of anticancer drugs. Considering their advantages, characteristics, and high efficacy, it was suggested that GFT be formulated in SLNs and their efficacy evaluated. In this study, four batches of GFT-loaded SLNs were developed, and their particle size, PDI, ZP, and drug encapsulation were evaluated. MTT assay revealed that optimized GFT-loaded SLNs (F4) exert significant cytotoxicity on MCF-7 breast cancer cells. Overall, our results indicate that SLNs may use p53, caspase-3, and caspase-9 pathways to trigger apoptosis in MCF-7 cells. The study therefore concludes that GFT-loaded SLNs are promising chemotherapeutics for the treatment of breast cancer. However, further pre-clinical and clinical evaluations are needed on the findings of this study.

## Figures and Tables

**Table 1:** Particle characterization of developed GFT-loaded SLNs.

GFT-Loaded SLNs	Particle Size (nm)	PDI	ZP (mV)	EE (%)
F1	378 ± 7.4	0.162 ± 0.002	−10.5 ± 1.3	64.07 ± 4.8
F2	405 ± 8.3	0.328 ± 0.014	−12.8 ± 1.2	66.67 ± 3.8
F3	465 ± 6.3	0.223 ± 0.011	−14.4 ± 0.8	71.69 ± 7.6
F4	472 ± 7.5	0.249 ± 0.004	−15.2 ± 2.3	83.18 ± 4.7

PDI, ZP, and EE stand for polydispersity index, zeta potential, and entrapment efficiency, respectively. “Particle size—significant difference (*p* < 0.0001) among all SLNs except F3 vs. F4 (non-significant); PDI—significant difference (*p* < 0.0001) among all SLNs; Zeta potential—non-significant among all SLNs except F1 vs. F4 (significant) (*p* < 0.05); EE%—significant difference (*p* < 0.05) between F1 vs. F4 and F2 vs. F4 and non-significant difference among F1 vs. F2, F1 vs. F3, F2 vs. F3, and F3 vs. F4. One-way ANOVA and Tukey’s multiple comparison between formulations are used to test these findings”.

**Table 2:** Results of particle characterization of GFT-loaded SLNs (F4) after stability study.

Months	Conditions	Size (nm ± SD)	PDI (±SD)	ZP (mV) (±SD)	EE (% ± SD)
0	-	473 ± 7.5	0.249 ± 0.004	−15.7 ± 2.3	83.2 ± 4.7
1	25 °C	474 ± 7.7	0.256 ± 0.009	−15.4 ± 2.0	82.3 ± 1.8
2		481 ± 2.6	0.266 ± 0.009	−14.5 ± 1.1	80.3 ± 3.2
3		488 ± 2.7	0.273 ± 0.005	−13.9 ± 5.5	79.2 ± 1.9
1	37 °C	490 ± 8.5	0.263 ± 0.036	−14.3 ± 0.6	79.3 ± 1.0
2		496 ± 8.0	0.281 ± 0.006	−14.2 ± 0.5	78.9 ± 2.3
3		500 ± 10.0	0.278 ± 0.005	−14.0 ± 1.8	77.8 ± 1.8

**Table 3:** Developed GFT-loaded SLN composition.

GFT-Loaded SLNs	GFT (mg)	Dynasan 114 (mg)	Lipoid 90H (mg)	Kolliphore^®^ 188 (%*w*/*v*)
F1	100	100	100	0.5
F2	100	100	150	0.5
F3	100	100	200	0.5
F4	100	100	250	0.5

## Data Availability

The data presented in this study are available on request from the corresponding author.
